# Effects of UVB radiation on grazing of two cladocerans from high-altitude Andean lakes

**DOI:** 10.1371/journal.pone.0174334

**Published:** 2017-04-05

**Authors:** Carla Eloisa Fernández, Danny Rejas

**Affiliations:** 1 Unidad de Limnología y Recursos Acuáticos, Universidad Mayor de San Simón, Cochabamba, Bolivia; 2 Division of Water Resources Engineering, Lund University, Lund, Sweden; VIT University, INDIA

## Abstract

Climate change and water extraction may result in increased exposition of the biota to ultraviolet-B radiation (UVB) in high-altitude Andean lakes. Although exposition to lethal doses in these lakes is unlikely, sub-lethal UVB doses may have strong impacts in key compartments such as zooplankton. Here, we aimed at determining the effect of sub-lethal UVB doses on filtration rates of two cladoceran species (*Daphnia pulicaria* and *Ceriodaphnia dubia*). We firstly estimated the Incipient Limiting Concentration (ILC) and the Gut Passage Time (GPT) for both species. Thereafter we exposed clones of each species to four increasing UVB doses (treatments): i) DUV-0 (Control), ii) DUV-1 (0.02 MJ m^2^), iii) DUV-2 (0.03 MJ m^2^) and iv) DUV-3 (0.15 MJ m^2^); and estimated their filtration rates using fluorescent micro-spheres. Our results suggest that increasing sub-lethal doses of UVB radiation may strongly disturb the structure and functioning of high-altitude Andean lakes. Filtration rates of *D*. *pulicaria* were not affected by the lowest dose applied (DUV-1), but decreased by 50% in treatments DUV-2 and DUV-3. Filtration rates for *C*. *dubia* were reduced by more than 80% in treatments DUV-1 and DUV-2 and 100% of mortality occurred at the highest UVB dose applied (DUV-3).

## Introduction

Depletion of the ozone layer and consequent increases in ultraviolet radiation (UVR) triggered the interest on the harmful effects of UVR on organisms, such as denaturation of proteins [1] and DNA damages [2–4]. Although the ozone layer depletion has stabilized, the effects on UVR will remain even by the end of this century [5, 6]. High-altitude Andean lakes ecosystems are particularly vulnerable, because of the extremely high levels of UVR occurring in this region [7], and the increased UVR penetration predicted for these environments due to indirect effects of climate change [7, 8].

The Andean region is characterized by the presence of numerous mountain lakes, mostly of glacial origin [9]. Underground connections and soil temperatures of the Central Andes (below 15°C but above freezing point) allow the flow of mobile forms of carbon, from the carbon-rich soils (239 to 466 Tm·ha^-1^; [10]) towards the lakes [11]. According to climate change scenarios, high-altitude Andean mountain ecosystems will experience warmer and drier climates in the future [7, 12, 13]. As a consequence of climate change, soil water saturation will decrease, leading to reduced concentrations of dissolved organic carbon (DOC) in lake waters, DOC being the most important UVR attenuation factor [14–16]. Hence, as an indirect consequence, climate change will likely lead to increasing exposure of aquatic organisms to UVR. Moreover, the rising need for water will further increase the exposure of organisms to UVR due to lower water levels in the lakes.

UVB radiation is the most harmful portion of the UVR spectrum for aquatic organisms [17]. UVB effects on zooplankton have been amply studied in rotifers [18, 19], copepods [20–22] and cladocerans [23–26]. For all of them, UVB radiation acts as a considerable stressor leading to reduced survival [23, 27] and, limited reproduction capacity [19]. UVB may also alter population dynamics [17, 28] and interactions between species [29], affecting therefore community composition and ecosystem dynamics [30].

Due to their location and normally high water transparency, it is assumed that aquatic biota from high-altitude Andean lakes is exposed to extremely high UVB irradiances [31]. However, zooplankton organisms employ several behavioral and physiological strategies to limit UVR damage. These strategies include vertical migration [17, 32, 33], production/accumulation of photo-protective compounds [34–36] and DNA damage reparation by photo-enzymatic processes [37–39]. Moreover, it has been shown that UVB is quickly attenuated in the water column in several lakes located in the Bolivian Central Andes, where attenuation depths (Z1%; the depth at which surface irradiance has decreased to 1%) range between 0.32 m and 1.07 m [40]. Therefore, it is unlikely that increased UVB doses will affect zooplankton mortality rates dramatically in nature. Sub-lethal damages, on the other hand, are likely more important than lethal effects, and may affect zooplankton in more subtle ways reducing their fitness [24, 25, 41].

High-altitude Andean lakes are characterized by simple trophic structures [42] making them very sensitive to environmental perturbations [43]. Naturally fishless, these systems are mainly regulated by zooplankton communities, mostly cladocerans (e.g. *Daphnia*, *Ceriodaphnia* and *Simocephalus*) and copepods (e.g. *Boeckella*). Despite the key role of zooplankton grazing in regulating phytoplankton productivity and energy transfer, studies on sub-lethal effects of UVR on zooplankton grazing activity are scarce and mostly concerned marine taxa [20, 44]. Nevertheless, these studies indicate that zooplankton grazing may be impaired by sub-lethal doses of UVR. The goal of this study was to evaluate (by using fluorescent particles) the potential effect of sub-lethal doses of UVB radiation on filtration rates of two cladoceran species (*Daphnia pulicaria* and *Ceriodaphnia dubia*) that are common and widely distributed in high-altitude Andean lakes [45, 46].

When performing zooplankton grazing experiments with fluorescent particles as markers, care must be taken to ensure that: (1) ingestion rates are not inhibited by high food concentrations (i.e. food concentrations are below the Incipient Limiting Concentration—ILC; defined as the concentration above which ingestion rates are physiologically and mechanically inhibited [47]), and (2) ingested particles are not excreted after passing through the digestive tract (i.e. feeding times are below the Gut Passage Time—GPT; defined as the time for food passing through the whole intestine [48–50]). In order to avoid these problems, estimations of ILC and GPT are essential. Thus, for both species the objectives were (1) to estimate the ILC, (2) to estimate the GPT, and (3) to test the effect of three doses of UVB on grazing activity by measuring filtration rates with fluorescent microspheres after exposure to UVB.

## Methods

ULRA/UMSS is an Authorized Scientific Institution (ICA), accreditation granted by the Bolivian Direction of Biodiversity and Protected Areas (DGBAP) to conduct biological scientific research within the Bolivian territory. Sampling activities were approved by administrative resolution VMABCC-026/09.

### Cladocerans origin and culture conditions

Zooplankton samples were collected in Totora-khocha Lake, located in The Central Andes in Bolivia (65.63° W– 17.46° S) at 3730 m above sea level, This is one of thousands of lakes formed during the last retreat of glaciers during the Pleistocene [9]. Air temperatures vary between 3.3 and 23.6°C during summer, and between -3.0 and 14.9°C during winter. Water temperature varies between 10.2 and 14.6°C. Mean and maximum depths of the lake are approximately 2.8 and 6.6 m, respectively. Secchi depth is approximately 1.6 m.

To collect the zooplankters, we took vertical and horizontal samples using a 100 μm mesh size plankton net. Captured individuals were gathered and transported alive to the laboratory in 2 L plastic bottles filled with lake water.

Approximately 100 individuals of *D*. *pulicaria* and *C*. *dubia* were isolated and acclimatized for 50–60 days to laboratory conditions (room temperature 20 ± 2°C, 13:11 light-dark cycles). Both species were fed with cultures of *Chlorella vulgaris*, supplying 25 μl of a 1.51·10^7^ ± 2.01·10^3^ cells mL^-1^ solution every day. After the adjustment period, adult females of each species were randomly selected to establish monoclonal cultures, from which we took the individuals for the experiments.

ILC and GPT are key parameters that depend on species body size and filtration capacities [51], thus, for both species we performed preliminary tests to determine concentration of microspheres, feeding times and range of food concentrations that allow properly counting each ingested microsphere and provide gross estimations of ILC and GPT. Based on those preliminary results, we designed more precise assays.

### Incipient limiting concentration

To measure ingestion rates, adult clones of both species were subjected to different food concentrations. Five feeding solutions for *D*. *pulicaria* (1·10^4^, 5·10^4^, 1·10^5^, 5·10^5^ and 1·10^6^ particles mL^-1^) and six for *C*. *dubia* (1·10^3^, 2.5·10^3^, 5·10^3^, 1·10^4^, 5·10^4^ and 1·10^5^ particles mL^-1^) were tested. Feeding solutions were composed by a mixture of *C*. *vulgaris*, in variable concentrations, and a fixed concentration of carboxylate fluorescent microspheres (3 μm ø Fluoresbrite^®^ YG—Polysciences, Germany); 1·10^3^ particles mL^-1^ for *D*. *pulicaria* and 3·10^3^ particles mL^-1^ for *C*. *dubia*. For each experimental unit (10 per treatment), one individual of *D*. *pulicaria* or *C*. *dubia* was placed in 5 mL of feeding solution for 10 or 4 minutes, respectively.

### Gut passage time

GPT of *D*. *pulicaria* and *C*. *dubia* were determined by placing adult clones individually in 5 mL of water (10 experimental units per treatment) with fluorescent microspheres at concentrations of 5·10^4^ particles mL^-1^ for *D*. *pulicaria* and 5·10^3^ particles mL^-1^ for *C*. *dubia*. Feeding solution concentrations were selected based on the ILC experiment results. Eight feeding times (1, 2, 3, 5, 10, 20, 40 and 60 min) were tested for *D*. *pulicaria*, and four feeding times (1, 3, 5 and 7 min) were tested for *C*. *dubia*.

Based on the results of the ILC and GPT assays (see Results section), we selected food concentrations lower than the ILC to prevent inhibition of ingestion rates; and feeding times shorter than the GPT to prevent excretion of particles during the experiments. Therefore, to perform filtration rate experiments we selected food concentrations of 5·10^4^ cells mL^-1^ and 5·10^3^ cells mL^-1^ for *D*. *pulicaria* and *C*. *dubia*, respectively. We established feeding times of 10 minutes for *D*. *pulicaria* and 4 minutes for *C*. *dubia*.

### UVB radiation and filtration rate experiments

To determine the effect of sub-lethal doses of UVB radiation on filtration rates, adult clones of each species were exposed to four treatments with increasing doses of artificial UVB radiation: *i)* DUV-0 (Control, no exposure to UVB), *ii)* DUV-1 (0.02 MJ m^2^), *iii)* DUV-2 (0.03 MJ m^2^) and *iv)* DUV-3 (0.15 MJ m^2^). Considering that daily UVB doses at the surface in the Central Andes in Bolivia range from 22 to 57 MJm^2^ [52] and attenuation coefficients (*Kd*) in the lakes vary from 4.29 to 11.73 [40], we estimated that UVB doses used in our experiments are expected to occur in nature between 0.6–1.7 m (DUV-1), 0.5–1.6 m (DUV-2) and 0.4–1.2 m (DUV-3).

Organisms of both species (14 experimental units per treatment) were placed individually in 5 cm depth transparent polycarbonate flasks with 10 mL of water. Then, they were irradiated with a 1.2 W lamp (PL-S 9W/01/2P Philips Electrical Ltd. Sywell, Northants, England). We employed a narrowband lamp (emission range of 305–315 nm and peak emission at 311 nm) to avoid short UVB and UVC wavelengths that UVB lamps usually emit, which are not found in sunlight and can be extremely damaging [53]. Samples were incubated at laboratory conditions. UVB exposure was applied once, in a 7 h interval within the light period. Exposure levels (doses) were applied varying the distance from the UVB source to the flasks. We performed filtration rate assays subsequently to one dark period (11h) after the UVB exposure. Organisms were placed individually in flasks with 5 mL of feeding solution. *D*. *pulicaria* were fed with a 5·10^4^ particles mL^-1^ solution (1·10^3^ particles mL^-1^ of fluorescent microspheres and 4.9·10^4^ cells mL^-1^ of *C*. *vulgaris*) for 10 minutes. *C*. *dubia* were fed with a 5·10^3^ particles mL^-1^ solution (3·10^3^ cells mL^-1^ fluorescent microspheres and 2·10^3^ cells of *C*. *vulgaris* mL^-1^) for 4 minutes.

### Laboratory procedures

At the end of all experiments, animals were anaesthetized with carbonated water to prevent microsphere excretion, and then, fixed with formaldehyde (2% final concentration). Each individual was first rinsed with deionized water to exclude microspheres that may be exteriorly adhered, placed in a glass slide and measured from the top of the helmet to the base of the tail. For the GPT assay, lengths of gut and food occupancy were measured in all individuals. For the remaining experiments, the organisms were dissolved individually with sodium hypochlorite to release the spheres from the digestive tract. All ingested microspheres were counted using epifluorescence microscopy (Olympus CX-31 microscope 400X; CX-RFL-2 UV light source; blue light).

### Data and statistical analysis

Filtration rate is defined as the volume of water separated from particulate matter by the filtering apparatus of one organism in a unit of time [54]. Ingestion and filtration rates were calculated for the ILC and UVB experiments by Eq (1):

FR=IRM (1)

where *FR* is the filtration rate (mL ind^-1^h^-1^), *IR* is the ingestion rate (calculated as the total number of food particles ingested in one hour) and *M* is the total amount of food particles in one milliliter of feeding solution.

After verifying assumptions of normality and homoscedasticity through diagnostic plots (Normal Q-Q and Residuals vs. Fitted, respectively) and Akaike Information Criteria (AIC), dependent variables of each experiment (relative food occupancy, ingestion or filtration rates) were compared among treatments by an Analysis of covariance (ANCOVA) and, when appropriate, orthogonal contrast post-hoc tests were applied. Size of each individual was used as covariate to correct for any effect of size on each dependent variable. All statistical analyses and figures were performed in R v3.2.2 [55].

## Results

### Incipient limiting concentration

**Fig 1:**
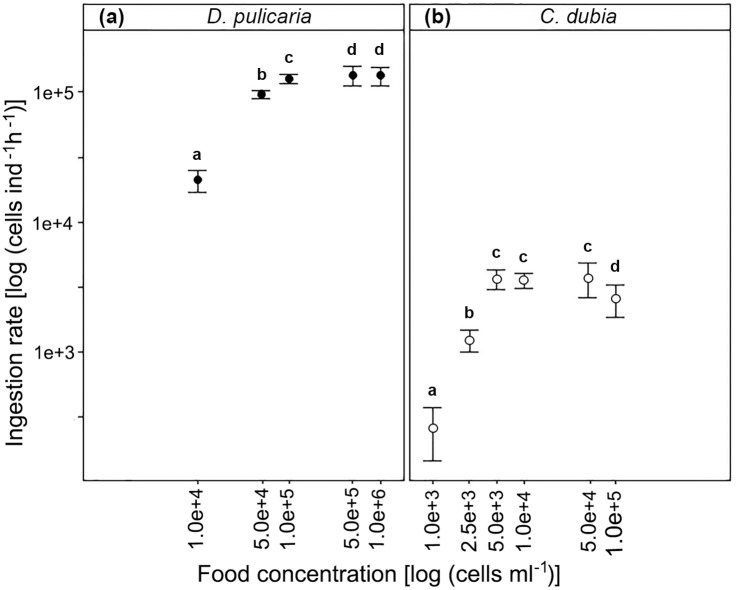
Ingestion rate in function of food concentration for (a) *D*. *pulicaria* and (b) *C*. *dubia*. Error bars correspond to two standard errors of the mean. Different letters indicate statistically significant differences between treatments (Contrasts post-hoc test). Note that both axes are at a logarithmic (log_10_) scale.

For both species, ingestion rates increased significantly at higher cell concentrations in the feeding solution (*D*. *pulicaria*, *F*_*4*,*44*_ = 41.7, *P* < 0.01; *C*. *dubia*, *F*_*5*,*52*_ = 19.4, *P* < 0.01; Fig 1). Maximum ingestion rates for *D*. *pulicaria* (1.4·10^5^ ± 1.1·10^4^ cells ind^-1^ h^-1^) occurred at a food concentration of 1·10^6^ cells mL^-1^. Ingestion rates at food concentrations of 1·10^6^ and higher were not statistically different (contrasts test, *P* > 0.05, Fig 1a). Therefore, we assumed the ILC for adult *D*. *pulicaria* (1468 ± 158 μm; mean length ± SD) feeding in a *Chlorella vulgaris* solution was 1·10^5^ cells mL^-1^, since ingestion rates were independent of the food concentration at higher food concentrations. This asymptotic effect of food concentration on ingestion rate, was also clear for *C*. *dubia* (961 ± 62 μm; Fig 1b). Maximum ingestion rates for *C*. *dubia* (3.7·10^3^ ± 5.5·10^2^ cells ind^-1^ h^-1^) were registered at a food concentration of 5·10^4^ cells mL^-1^. Ingestion rates did not increase significantly at food concentrations higher than 5·10^3^ cells mL^-1^ (contrasts test, *P*-values > 0.05, Fig 1b).

### Gut passage time

**Fig 2:**
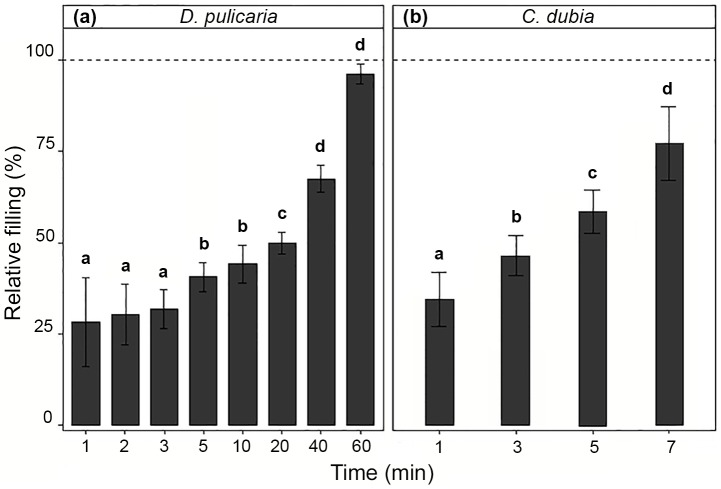
Relative gut filling in (a) *D*. *pulicaria* and (b) *C*. *dubia* as a function of feeding time. Feeding solution was a mix of *C*. *vulgaris* and fluorescent microspheres. Error bars correspond to twice the standard error of the mean. Different letters indicate statistically significant differences between treatments (Contrasts's post-hoc test).

Our results showed that GPT for *D*. *pulicaria* feeding in *C*. *vulgaris* solution was between 40 and 60 minutes. After 40 minutes of feeding, all individuals of *D*. *pulicaria* showed more than 76% of gut filling. After 60 minutes, 40% of the individuals of *D*. *pulicaria* showed complete gut filling. *C*. *dubia* exhibited GPT’s between 5 and 7 minutes. All individuals of *C*. *dubia* showed more than 77% of gut filling after 5 minutes of feeding, whereas 20% of the individuals showed complete filling after 7 minutes of feeding (Fig 2).

### Effects of UVB radiation on filtration rates

**Fig 3:**
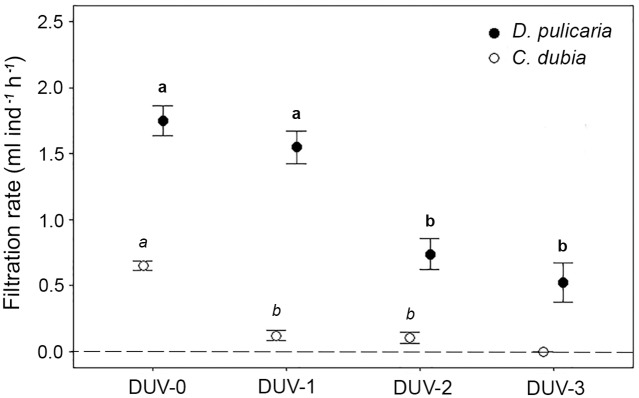
Filtration rates of *D*. *pulicaria* (black dots) and *C*. *dubia* (unfilled dots) exposed to sublethal UVB doses. DUV-0: no exposition, DUV-1: 0.02 MJ m^2^, DUV-2: 0.03 MJ m^2^ and DUV-3: 0.15 MJ m^2^. Error bars correspond to the standard deviation of the mean. Different letters indicate statistically significant differences between treatments (Contrasts's post-hoc test).

We found a strong effect of UVB radiation on filtration rates of both species (*D*. *pulicaria*, *F*_*3*,*41*_ = 22.5, *P* < 0.01; *C*. *dubia*, *F*_*2*,*36*_ = 73.2, *P* < 0.01; Fig 3) in our experiments. Filtration rates of *D*. *pulicaria* (1701 ± 152 μm) in treatment with the lowest UVB dose DUV-1 (1.55 ± 0.56 mL ind^-1^ h^-1^) were not significantly different from the control treatment DUV-0 (1.75 ± 0.34 mL ind^-1^ h^-1^; *post-hoc* test, *P* = 0.24). Filtration rates were significantly lower (*post-hoc* test, *P* < 0.001) in treatments DUV-2 (0.74 ± 0.40 mL ind^-1^ h^-1^) and DUV-3 (0.52 ± 0.18 mL ind^-1^ h^-1^) than in the control treatment. Filtration rates were not significantly different between treatments DUV-2 and DUV-3 (*post-hoc* test, *P* = 0.21).

Strong reductions in filtration rates of *C*. *dubia* (888 ± 50 μm) were detected even at the lowest UVB dose (ANCOVA, F_2,36_ = 73.16; *P* <0.01). Filtration rates were significantly lower in treatments DUV-1 (0.12 ± 0.05 mL ind^-1^ h^-1^) and DUV-2 (0.11 ± 0.07 mL ind^-1^ h^-1^) with respect to the control treatment (DUV-0; 0.64 ± 0.2 mL ind^-1^ h^-1^), representing reductions of 81 and 82% on filtration rates, respectively. Nevertheless, there were no significant differences between DUV-1 and DUV-2 (*post-hoc* test, *P* = 0.27). No individuals of *C*. *dubia* in DUV-3 survived the treatment.

## Discussion

In this study, we investigated experimentally the effect of increasing doses of UVB radiation on filtration rates of *D*. *pulicaria* and *C*. *dubia*. Incipient Limiting Concentration (ILC) and Gut Passage Time (GPT) were estimated previously as key parameters to determine optimum food concentrations (algal concentrations below the ILC) and feeding times (below the GPT). Compared to former studies, we found comparable ILC values for *D*. *pulicaria* and *C*. *dubia*. ILC values for cladocerans have been reported as low as 6·10^3^ cells _(*Oocystis sp*.)_ mL^-1^ for *Moina micrura* and as high as 4.2·10^5^ cells _(*Chlorella sp*.)_ mL^-1^ for *D*. *ambigua* [50]. Our estimation of the ILC for *D*. *pulicaria* (1·10^5^ cells _(*C*. *vulgaris*)_ mL^-1)^ falls well within this range. The small *C*. *dubia* (mean length 961 μm) showed lower ILC (5·10^3^ cells _(C. vulgaris)_ mL^-1^) than the larger *D*. *pulicaria* (mean length 1468 μm). Low ILC values are expected for populations adapted to oligotrophic conditions [56]. The pattern of higher feeding efficiency of larger species compared to smaller ones has been reported earlier [57, 58] and is mainly explained by the increasing energy demand with organism size [51, 59].

GPT values for *Daphnia* species vary in a wide range depending on the species (grazer and prey), method of estimation, size of the animals and food concentration [60–62]. GPT values between 25 and 54 minutes have been reported for different species of the genus *Daphnia* [49]. According to Wiedner and Vareschi [63], GPT’s are shorter at higher food concentrations, and feeding times in laboratory experiments with *D*. *pulex* should not exceed 2 minutes when using food concentrations of 10^5^ cells mL^-1^ or higher. However, GPT estimations for *D*. *pulicaria* show strong variations, ranging between 4 and 106 minutes even for the same prey species and same conditions [62, 64]. Hence, our GPT estimation for *D*. *pulicaria* (40–60 minutes) is within the range previously found. To our knowledge, GPT values for *C*. *dubia* have not previously been assessed. However, food evacuation rates tend to be lower in small cladocerans than in large ones [49]. GPT values for chydorids, similar in size to *C*. *dubia* (< 1 mm), range between 5 to 7 minutes [65, 66]. These results match exactly with our GPT estimate for *C*. *dubia*.

It has been demonstrated that UV radiation affects cladocerans in several ways, such as reducing the number of offspring [24, 67, 68], changing swimming behavior [32, 69, 70], and causing visceral [71] and DNA damage [68, 72]. Some metabolic and physiological effects of sub-lethal UV doses on cladoceran species have also been noted, such as decreased/increased respiration rates [73], reduced activities of digestive enzymes, and reduced residence time of algae in the gut [41]. Our study is the first to demonstrate a negative effect of sub-lethal UVB doses on the filtration rate of cladocerans. We found that filtration rates of *D*. *pulicaria* decreased to less than 50% in individuals exposed to naturally occurring doses of UVB radiation. According to data from 14 lakes in The Central Andes of Bolivia [40], doses in treatments DUV-2 and DUV-3 are expected to occur at maximum depths of 1.6 m. Hence, it is important to highlight that diel vertical migration may play a key role in avoiding UVB radiation [28, 74, 75]. Our findings suggest that if *D*. *pulicaria* remains at depths shallower than 1.6 m, filtration rates could be strongly affected. While it was expected that higher doses result in stronger effects [76], we found no further reduction in filtering rates of *D*. *pulicaria* in treatment DUV-3 (0.15 MJ m^2^ d^-1^; corresponding to 1.2 m depth) compared to treatment DUV-2 (0.03 MJ m^2^ d^-1^; corresponding to 1.6 m depth). Filtration rates in treatment DUV-1 (0.02 MJ m^2^ d^-1^; corresponding to 1.7 m depth) were not statistically different from the control treatment DUV-0. It is unlikely that animals get exposed to higher doses under natural conditions, which means that individuals that are able to avoid the first 1.6 m of the water column during the daytime, are likely to be unaffected by UVB radiation.

The effect of UVB radiation on *C*. *dubia* was even stronger; treatment DUV-3 (0.15 MJ m^2^ d^-1^) caused 100% mortality, whereas filtration rates were reduced to less than 20% in treatments DUV-1 (0.02 MJ m^2^ d^-1^) and DUV-2 (0.03 MJ m^2^ d^-1^). High mortality of *C*. *dubia* was also previously observed in southern Chilean wetlands when UVB doses exceeded 0.07 MJ m^2^ d^-1^ [77]. Our results, together with field observations describing *C*. *dubia* as an unpigmented species [78] that commonly avoids the upper surface waters in transparent lakes even in absence of predators [79, 80], suggest that *C*. *dubia* must remain deep in the water column to prevent lethal effects. The depth necessary for *C*. *dubia* to prevent the effects of UVB radiation will depend on the attenuation coefficient, being around 1.7 m in the region from where the organisms were collected [40].

A huge variation in tolerance to UVB radiation has been reported for cladocerans. Some studies suggest that small cladocerans are more tolerant to UVB radiation than larger ones [80]. Extremely low tolerance was reported for the large *Daphnia magna* (lethal doses LD_50_ 0.013 MJ m^2^; [27], in comparison with smaller species such as *C*. *dubia* (LD_50_ = 0.035 MJ m^2^) and *Bosmina meridionalis* (LD_50_ = 0.04 MJ m^2^; [81]. This pattern may occur due to the low energy cost that repair mechanisms represent for small-sized organisms [82, 83]. A similar pattern was described between individuals of the same species for *Daphnia galeata* and *D*. *pulex*, probably due to the transfer of photoprotective pigments from the mother [84–86]. However, and in accordance with results of our study, the reverse pattern has also been found: UVB tolerance of the large *Daphnia pulex* (LD_50_ = 0.06 MJ m^2^; [87] was found to double that of the small species *C*. *dubia* (LD_50_ = 0.035 MJ m^2^; [81]. These contrasting results suggest that factors other than body size may also play a role in UVB tolerance for cladocerans. The evidence that photoprotective pigments provide tolerance to some species [15, 36, 87], argue for the need of further studies to explain tolerance to UVB radiation.

There is a positive correlation between cladocerans size and filtration rates [54, 57, 58]. Large species are more efficient grazers, and their impact on the energy flux in aquatic ecosystems is disproportionately high compared to that of small species. Top-down control on phytoplankton is (co)determined by the proportional relationship between large and small zooplankton species [58]. Even if *D*. *pulicaria* was less affected by UVB than *C*. *dubia*, a 50% reduction of filtration rates of *D*. *pulicaria* may have stronger impact on the overall zooplankton community grazing rates than an 80% reduction in the filtration rates of *C*. *dubia*. Indeed, a reduction of zooplankton grazing pressure decreases the zooplankton/phytoplankton biomass ratio [88], leading in a break up in the energy flux.

Our results indicate that UVB radiation is a strong selective force in high-altitude Andean lakes, which excludes grazers from utilizing resources in surface waters during daytime. Climate change, through an indirect increase in UVB radiation, and water extraction, through a reduction in water depth, will strongly reduce zooplankton grazing and ultimately the energy flux in these ecosystems. This could lead to increase the risk of algal blooms, even if this risk is currently low due to nutrient limitation [89] and to consequent decrease in water quality for human uses.
